# Case Report: THSD7A-Positive Membranous Nephropathy Caused by Tislelizumab in a Lung Cancer Patient

**DOI:** 10.3389/fimmu.2021.619147

**Published:** 2021-05-10

**Authors:** Minjiang Chen, Lei Zhang, Wei Zhong, Ke Zheng, Wei Ye, Mengzhao Wang

**Affiliations:** ^1^ Department of Respiratory and Critical Care Medicine, Peking Union Medical College Hospital, Beijing, China; ^2^ Department of Nephrology, Peking Union Medical College Hospital, Beijing, China

**Keywords:** immune checkpoint inhibitors, immune related adverse event, membranous nephropathy, non-small cell lung cancer, THSD7A (thrombospondin type 1 domain-containing protein 7A)

## Abstract

Immune checkpoint inhibitors (ICIs) became the standard treatment for many different kinds of cancers and can result in a variety of immune-related adverse events (irAEs). IrAEs of kidney are uncommon and consists of different pathology types. Among the different types, membranous nephropathy (MN) is rare and have not been well-described. Since MN can also be associated with malignancies, differential diagnosis in patients receiving ICIs who develop MN can be very difficult. We present the case of a 74-year-old man with metastatic non-small cell lung cancer who developed MN after ICIs therapy. The patient tested positive for thrombospondin type-1 domain-containing 7A antibodies (THSD7A) when diagnosed with MN. Supplementary examinations revealed the predisposing antigen in the primary tumor and present of the antibody after immunotherapy, which corresponded to the patient’s clinical course of nephropathy. Treatment consisting of systemic glucocorticoids and rituximab resulted in a good clinical response, and the THSD7A antibodies were no longer detected. In this case, we first discuss the potential mechanism of immunotherapy related MN, in which the activation of humoral immunity may play an important role.

## Introduction

The use of immune checkpoint inhibitors (ICIs) caused a variety of immune-mediated adverse events (irAEs). The underlying mechanism includes an increasing T cell activity and autoimmune antibodies (1). Kidney irAEs, albeit uncommon, is being increasingly recognized with the expanded ICIs use (2, 3). Membranous nephropathy (MN) has rarely been reported and the underlying mechanism remains unclear.

Herein, we describe an interesting MN case with non-small cell lung cancer after tislelizumab (a PD-1 inhibitor) (4) treatment. In particular, this patient tested positive for THSD7A antibodies, which was rare and had been proven to play an important role in the development of MN (5, 6). In this case report, we described the changes in autoimmune antibodies in during the development and remission of nephropathy and highlight the possibility of humoral immunity activation as a pathogenic mechanism in ICI-related MN.

## Case Report

**Figure 1:**
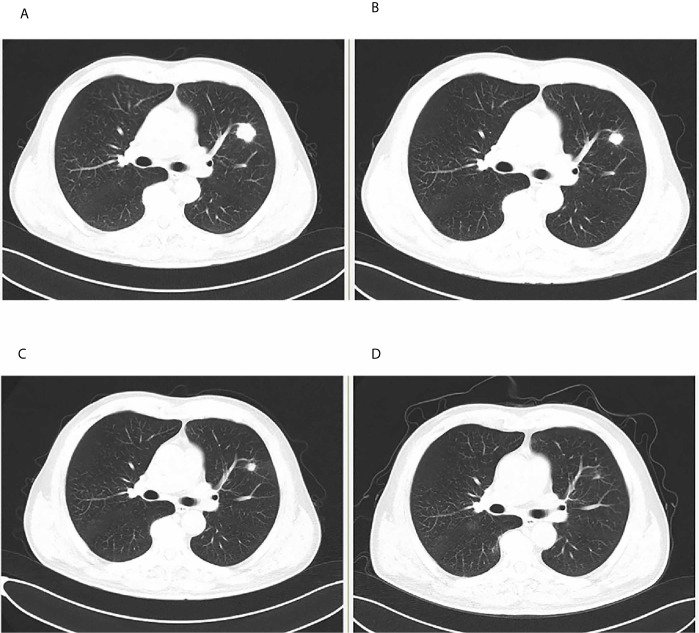
Tumor assessment during tislelizumab treatment. **(A)** Baseline before treatment. **(B)** After two cycles of induction treatment. **(C)** After four cycles of induction treatment. **(D)** At membranous nephropathy diagnosis.

A 74-year-old man with metastatic lung adenocarcinoma and no history of chronic renal disease enrolled in the BGB-A317-304 open labeled trial (NCT03663205) on June 25, 2019. His baseline urine protein and serum albumin and creatinine levels were within the normal range (**Table 1**). He was randomly categorized into the immunotherapy group and was initially treated with a tislelizumab and chemotherapy combination that included pemetrexed and carboplatin for 4 cycles, followed by maintenance therapy with tislelizumab and pemetrexed for 11 cycles until April 23, 2020. Partial response was achieved and persisted after 2 cycles (**Figure 1**). However, the patient experienced fatigue and chronic onset of mild edema of both lower extremities from late April. On May 7, laboratory findings revealed a decrease in serum albumin level to 19 g/L, and a substantial increase in 24-hour urine protein level to 20.16 g. Serological markers of MN, THSD7A and antigen phospholipase A2 receptor 1 (PLA2R1) antibodies were also tested using a cell based indirect immunofluorescence assay (7). The results were negative for PLA2R1 antibodies and positive for THSD7A with a titer of 1:100. Nephrotic syndrome was diagnosed, and the patient was referred to the nephrology department.

**Table 1:** Laboratory values and treatment timeline.

Date	WBC (10^9^/L)	HGB (G/L)	PLT (10^9^/L)	ALB (G/L)	Cr (μmol/L)	Urea (mmol/L)	K (mmol/L)	Na (mmol/L)	Ca (mmol/L)	Urine protein (g/L)	24h Urine protein (g)	THSD7A antibodies (titer)	Treatment and events timeline
June 19, 2019	6.00	153	169	41	67	5.38	4.3	140	2.24	Negative	NE	Negative	Baseline before treatment
September 20, 2019	5.02	129	199	49	69	5.07	4.3	139	2.44	Negative	NE	Negative	Four cycles of induced treatment
December 12, 2019	5.41	128	213	43	64	3.94	4.3	141	2.21	Negative	NE	1:100	Four cycles of maintenance therapy
May 7, 2020	5.97	137	176	19	82	4.72	3.9	139	1.99	≥3.0	20.16	1:100^#^	Eleven cycles of maintenance therapy; Nephrotic syndrome
May 14, 2020	5.97	124	170	20	78	6.65	3.4	141	1.98	≥3.0	11.63	NE^*^	Renal Biopsy; Glucocorticoids administered
May 27, 2020	13.95	142	176	26	84	7.17	4.3	139	2.11	1.0	9.85	NE	Rituximab administered
July 13, 2020	9.25	136	161	36	62	5.22	3.8	141	2.22	0.3	2.53	Negative	Two months after therapy; Prednisone tapered
Nov 30,2020	7.48	144	145	44	63	4.91	4.0	141	2.43	Negative	0.59	NE	Prednisone stopped
Feb 22, 2021	5.58	149	127	38	57	6.62	4.4	140	2.24	Negative	0.17	Negative	Follow-up visit

*NE: not evaluated; ^#^the THSD7A antibodies were tested on May 12, 2020.

**Figure 2:**
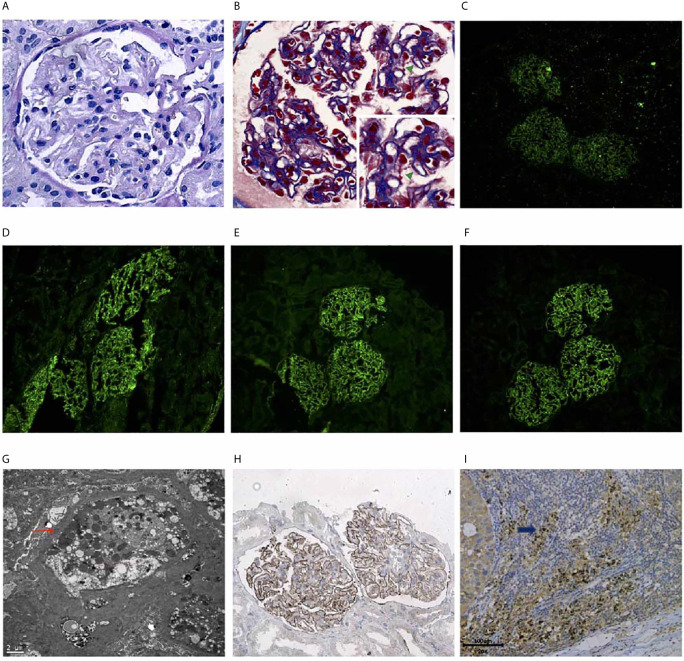
Renal biopsy findings showing a THSD7A-associated MN Renal histology specimens and baseline tumor tissue. **(A)** Periodic acid-Schiff (PAS) stain showing stiff glomeruli (Original magnification, 400×). **(B)** Masson trichrome stain showing subepithelially localized immune deposits (green arrow) (Original magnification, 400×). **(C)** Immunofluorescence of IgG deposition in the subepithelial area. (Original magnification, 200×). **(D)** Immunofluorescence of subepithelial IgG1 deposition. (Original magnification, 200×). **(E)** Immunofluorescence of subepithelial IgG2 deposition. (Original magnification, 200×). **(F)** Immunofluorescence of subepithelial IgG4 deposition. (Original magnification, 200×). **(G)** Electron microscopy showing discrete electron-dense subepithelial deposits (red arrow). (Original magnification, 6000×). **(H)** Positive staining for thrombospondin type-1 domain-containing 7A (THSD7A) along the glomerular basement membrane. (Original magnification × 200). **(I)** The tumor cells of baseline metastases lymph node were positive for thrombospondin type-1 domain-containing 7A (THSD7A) by immunohistochemistry (blue arrows).

Renal biopsy was performed. Light microscopy showed stiffness in the glomeruli with scattered subepithelially localized immune deposits (Masson stain) containing slightly focal tubular atrophy and interstitial fibrosis, consistent with early MN (**Figures 2A, B**). Immunofluorescence staining showed granular immunoglobulin G (IgG) deposits (**Figure 2C**), including IgG1, IgG2 and IgG4, uniformly and subepithelially distributed in the glomeruli (**Figures 2D–F**). The immunofluorescence staining of IgG3 was negative. Electron microscopy showed discrete electron-dense deposits at the subepithelial surface of the glomerular capillary wall, accompanied by effacement of overlying epithelial cell foot processes (**Figure 2G**). Immunohistochemical analyses revealed positive staining for THSD7A along the glomerular basement membrane (**Figure 2H**).

The differential diagnosis during renal biopsy was nephrotic syndrome either due to tislelizumab treatment or as a paraneoplastic sign. Determinate when the THSD7A antibodies appeared helped in distinguishing between the two different pathogenies.

**Figure 3:**
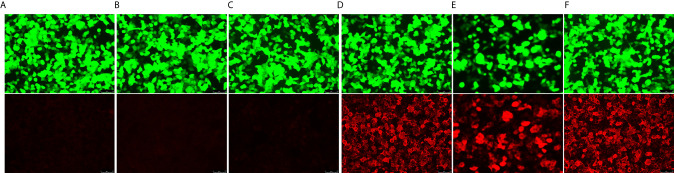
Series anti-thrombospondin type-1 domain-containing 7A (THSD7A) antibody tests. THSD7A IgG detected in the serum using cell-based indirect immunofluorescence test. Images on the top show transfection cells express green fluorescent protein. The images on the bottom show transfected cells with the red fluorescent-labeled secondary antibody on detection of anti-THSD7A IgG. **(A)** Negative control. **(B)** Baseline before treatment (June 19, 2019). **(C)** After four cycles of induced treatment (September 20, 2019). **(D)** After four cycles of maintenance therapy (December 12, 2019). **(E)** At nephrotic syndrome diagnosis (May 12, 2020). **(F)** Positive control.

The THSD7A tumor antigen and antibodies against it were tested using archived tumor and consecutive serum specimens. The baseline tumor tissue tested positive for the THSD7A antigen tested positive (**Figure 2I**). The patient tested negative for THSD7A antibodies at baseline but tested positive after 4 cycles of maintenance tislelizumab therapy (**Figure 3**). Therefore, MN was thought to be related with the ICI treatment.

Tislelizumab was discontinued following the diagnosis of nephropathy. To treat biopsy-proven MN, intravenous methylprednisolone (60 mg) was administered for 14 consecutive days, followed by oral prednisone (60 mg) once daily. Rituximab (1 g) was also administered once at the beginning of treatment. Two months after initiating glucocorticoid therapy, the 24-hour urine protein level decreased to 2.53 g and the serum albumin level improved to 36 g/L (**Table 1**). The serum also tested negative for THSD7A antibodies. Consequently, treatment with prednisone was slowly tapered. After a total course of about six months the systemic glucocorticoids were stopped at the end of November 2020. The patient was followed-up till the March 2021. His laboratory test including serum albumin, creatinine levels, and urinalyses were all in the normal range (**Table 1**). He did not receive any antitumor therapy after MN. CT scans including chest and abdomen were regularly performed, which showed persistent partial response of lung cancer.

## Discussion

Renal immune-mediated adverse events (irAEs), which had different clinical and histological manifestations, have not been commonly reported in previous studies (2). Among the different types of renal irAEs, acute interstitial nephritis characterized by diffuse interstitial inflammation with a predominant T-lymphocytic infiltrate (3) was the most common. Glomerular was less affected by immunotherapy, and pauci-immune glomerulonephritis, podocytopathies, and complement 3 glomerulonephritis are the most frequently reported histology subtypes (8). MN is an antibody-mediated autoimmune glomerular disease (9) that typically present with marked elevation in urine protein levels and decline in serum albumin levels and is rarely reported to be associated with ICIs therapy (10). In patients with underlying cancers, MN was also considered to be a paraneoplastic sign, especially if THSD7A antibodies were present (6, 11). To our knowledge, this is the first report of THSD7A-positive MN related with ICIs therapy.

THSD7A, which is also expressed in various tumors, is the target podocyte antigen identified in MN (12). A potential mechanism for the association between cancer and MN with respect to the THSD7A antibodies has been described (5). However, with the commonly present antigens in tumors, the THSD7A antibodies are rare in patients with cancer prior to treatment (11), as shown in this case. Moreover, paraneoplastic glomerular diseases often appeared when cancer is activated or recurrent (13, 14) and should be achieved with treatment of the underlying cancer.

In our patient, despite the redisposing THSD7A antigen in the primary tumor, the THSD7A antibody tested positive after immunotherapy, and symptomatic nephropathy subsequently developed while the tumor was still in remission. After systemic glucocorticoids and rituximab treatment, the patient tested negative for the antibodies together with the remission of nephropathy. Because of the timeline of ICIs therapy, MN, and mismatched kidney disease with tumor response, we speculated that tislelizumab-associated humoral immunity activation, which leads to an increase of THSD7A antibody titer or an *de novo* production of THSD7A antibodies, may have contributed to the development of renal irAEs, as observed in our case.

While a T cell-mediated mechanism is considered the predominant mechanism for irAEs, it is increasingly recognized that humoral immunity may also play an important role in irAEs (15). Similar autoantibodies have also been reported for different irAEs such as bullous pemphigoid and myasthenia gravis with their autoimmune disease counterparts (16, 17). Reactivation of some previously unrecognized antibodies were also found in some irAEs (18). Moreover, well-controlled pre-existing autoimmune or antibody-mediated diseases, such as PLA2R antibody-positive primary MN, could also be reactivated during ICI therapy (19, 20).

Based on the above information, we hypothesized that anti-CD20 antibodies may be effective in the treatment of irAEs. In this case, the patients’ MN was thought to be mediated by humoral immunity and had been well-controlled by prednisone and rituximab (a monoclonal anti-CD20 antibody). This suggested us that the treatment for irAEs could be selected according to the different underlying mechanisms.

## Conclusion

MN is a rare renal manifestation associated with ICIs. The underlying mechanism likely involves the production of podocyte antibodies including THSD7A antibodies. This case demonstrated that similar autoantibodies may be present in cases of immune-related glomerular diseases and may also have a similar mechanism with idiopathic MN, in which the humoral immunity may play an important role. A better understanding of the underlying mechanism might be useful in monitoring and individualized treatments of irAEs.

## Data Availability Statement

The original contributions presented in the study are included in the article/supplementary material. Further inquiries can be directed to the corresponding authors.

## Ethics Statement

The studies involving human participants were reviewed and approved by The studies involving human participants were reviewed and approved by Center for Ethics in Peking Union Medical College Hospital. The patients/participants provided their written informed consent to participate in this study. Written informed consent was obtained from the individual(s) for the publication of any potentially identifiable images or data included in this article.

## Author Contributions

MC analyzed the patient data, designed the case report, and drafted the manuscript. WZ provided significant contributions to the collection of patient data and sample preparation. WY and LZ performed the renal biopsy and provided the renal pathology images. KZ and MW provided significant contributions to the analysis of the patient data and designed the case report. All authors contributed to the article and approved the submitted version.

## Funding

This work was supported by the National Natural Science Foundation (Grant No. 81702292).

## Conflict of Interest

The authors declare that the research was conducted in the absence of any commercial or financial relationships that could be construed as a potential conflict of interest.
